# Quantifying neuroinflammation within deep gray matter in small vessel disease using diffusion tensor based free-water imaging: a longitudinal study

**DOI:** 10.3389/fnagi.2024.1361436

**Published:** 2024-07-10

**Authors:** Yawen Sun, Xu Han, Zhenghao Li, Yage Qiu, Ying Hu, Yuyao Zhang, Yongming Dai, Hongjiang Wei, Qun Xu, Yan Zhou

**Affiliations:** ^1^Department of Radiology, Ren Ji Hospital, School of Medicine, Shanghai Jiao Tong University, Shanghai, China; ^2^School of Biomedical Engineering, Shanghai Jiao Tong University, Shanghai, China; ^3^School of Information and Science and Technology, ShanghaiTech University, Shanghai, China; ^4^School of Biomedical Engineering and State Key Laboratory of Advanced Medical Materials and Devices, ShanghaiTech University, Shanghai, China; ^5^Department of Health Manage Center, Ren Ji Hospital, School of Medicine, Shanghai Jiao Tong University, Shanghai, China; ^6^Department of Neurology, Ren Ji Hospital, School of Medicine, Shanghai Jiao Tong University, Shanghai, China

**Keywords:** small vessel disease, free-water imaging, deep gray matter, neuroinflammation, cognitive function

## Abstract

**Purpose:**

Employing free water (FW) imaging, a cutting-edge diffusion MRI technique, we assessed neuroinflammation within deep gray matter (DGM) in small vessel disease (SVD) over 1–2 years.

**Method:**

One hundred and seventy SVD patients and 21 healthy controls (HCs) underwent MRI scans and neuropsychological evaluations at baseline. These patients were then categorized into two groups: 67 displayed no cognitive impairment (NCI), while 103 exhibited vascular mild cognitive impairment (VaMCI). A follow-up study 1–2 years later included 23 from the NCI group and 28 from the VaMCI group. Calculation of FW values within DGM facilitated both cross-sectional and longitudinal analysis, revealing partial correlations between FW value changes and cognitive function alternations.

**Results:**

Baseline examinations disclosed significant differences in DGM FW values among the three participant groups. We found increased mean FW values in the left pulvinar (Pul), bilateral lateral nuclei (LN) and bilateral internal medullary lamina of the thalamus in VaMCI participants compared with their NCI counterparts in longitudinal analysis. Notably, negative associations emerged between the FW value changes in the left Pul and the right LN of the thalamus and MoCA score changes in the VaMCI group over 1–2 years.

**Conclusions:**

These findings support the hypothesis that increased FW value is present at the preclinical stage of SVD and remains persistent during the early course of the disease, potentially acting as the biomarker for the mechanism of underlying cognitive decline in SVD.

## 1 Introduction

As a prevalent etiological factor for dementia in the elderly, small vessel disease (SVD) accounts for ~45% of all dementia disorders (Wardlaw et al., 2013a). This disease typically evolves from a stage of no cognitive impairment (NCI), escalates to vascular mild cognitive impairment (VaMCI), and ultimately peaks in subcortical vascular dementia (SVaD). Early diagnosis of VaMCI, or even NCI, carries crucial clinical implications, as cognitive decline tends to be progressive and may become irreversible in the advanced stage of SVaD.

Recognized as key MRI indicators for SVD, white matter hyperintensities (WMHs) have been thoroughly validated (Wardlaw et al., 2013b). Along with adjacent normal-appearing white matter (NAWM), WHMs have demonstrated correlations with cognitive impairment in SVD (Brandhofe et al., 2021). While most SVD research has targeted white matter, emerging studies are exploring alterations in deep gray matter (DGM) regions—namely, basal ganglia and thalamus—which have association with both SVD progression and cognitive impairments (Liu et al., 2015; Bonifazi et al., 2018; Karim et al., 2020).

Chronic global hypoperfusion, a hallmark of SVD, renders DGM regions particularly vulnerable due to their dependence on deep penetrating arteries, leading to progressive neurodegeneration accompanied by robust inflammation (Cao et al., 2021). This inflammation not only accelerates SVD but also triggers iron deposition, amplifying free-radical damage (Andersen et al., 2014; Low et al., 2019). Moreover, it may also disrupt the blood-brain barrier (BBB), causing fluid leakage into perivascular tissues (Kerkhofs et al., 2021). Despite current limitations in pathological research, non-invasive MRI techniques offer a sensitive avenue for *in vivo* monitoring of these complex changes.

Fractional volume, a metric derived from diffusion tensor based Free-Water (FW) imaging, has been proposed to track changes in extracellular FW, thereby providing insights into underlying pathologies such as neuroinflammation (Pasternak et al., 2012a; Di Biase et al., 2020; Febo et al., 2020). Previous studies demonstrated increased FW values in white matter associated with SVD, suggesting the occurrence of tissue-based neuroinflammation (Duering et al., 2018; Mayer et al., 2022). Intriguingly, increased FW have also been identified as a robust predictor of cognitive function impairment (Gullett et al., 2020). However, few studies have been conducted to investigate FW content specifically within DGM regions in the context of SVD.

The goal of this study was to quantify the alterations in FW values within DGM during early SVD, and to investigate its correlation with cognitive function as a potential biomarker for monitoring SVD progression.

## 2 Materials and methods

### 2.1 Participants

The studies involving humans were approved by the Research Ethics Committee of the Ren Ji Hospital, School of Medicine, Shanghai Jiao Tong University. The studies were conducted in accordance with the local legislation and institutional requirements. The participants provided their written informed consent to participate in this study.

One hundred and seventy SVD patients were enrolled from the neurology department in our hospital. Each participant underwent a standard baseline evaluation including complete sociodemographic, neurological examination, clinical data, neuropsychological tests and multimodal brain MRI scan. All recruited participants met the following criteria: (i) 50–85 years old; (ii) education years ≥ 6 years; (iii) at least 1 month after clinical stroke accident; (iv) presence of ≥ 1 subcortical lacunar infarct(s) and white matter lesions on MRI; and (v) a modified Rankin score ≤ 3 points. The exclusion criteria were as follows: (i) severe brain atrophy; (ii) intracranial space-occupying lesions; (iii) hemorrhage, large infarcts or acute infarcts; (iv) lacunar infarct, calcification, microbleeds or hemorrhage in the DGM; (v) severe systemic or other diseases that may cause cognitive dysfunction; (vi) cardioembolic or large-vessel diseases; (vii) history of intracranial surgery or traumatic brain injury; (viii) severe depression; other major central neurological or psychiatric disorders; and (ix) claustrophobia or contraindications to MRI examination. Two experienced neuroradiologists were tasked with evaluating the MRI images. All the SVD patients were categorized into two groups: 103 patients with VaMCI and 67 patients with NCI. VaMCI was diagnosed according to the criteria for Vascular Behavioral and Cognitive Disorders (VASCOG) (Sachdev et al., 2014), which is in line with the Neurocognitive Disorders Work Group of the fifth revision of the Diagnostic and Statistical Manual of Mental Disorders (DSM-5). The VaMCI group was diagnosed with one or more impaired cognitive domains but remained independent regarding daily living ability. The NCI group was diagnosed with SVD patients whose cognitive functions were all within the normal range.

Twenty-one healthy participants from the Tangqiao community, Pudong New District in Shanghai, were enrolled as HCs. The following inclusion criteria for HC were applied: (i) 50–85 years old; (ii) education years ≥ 6 years; (iii) no history of clinical stroke or severe diseases for important organs; (iv) without other obvious structural abnormalities on MRI scans; (v) Montreal cognitive assessment (MoCA) and mini mental state examination (MMSE) were within the normal range; and (vi) no evidence of vascular risk factors.

Follow-up brain MRI scans and neuropsychological tests were performed 1–2 years later. No participant suffered a stroke or a transient ischemic attack during this time. The main treatment is to manage the risk factors for SVD according to clinical practice guidelines and healthy lifestyle behaviors. Finally, altogether 51 SVD patients with complete brain MRI scans and neuropsychological assessments both at baseline and follow-up were enrolled, including 28 VaMCI and 23 NCI patients.

### 2.2 Neuropsychological tests

All the participants underwent a standardized battery of multidomain cognitive tests within a week after the MRI scan by trained neuropsychologists. The following tests were used to evaluate 4 key cognitive domains: (i) attention and executive function: trail-making tests A and B (TMT-A, TMT-B), Stroop color-word test C (Stroop C-T) and category verbal fluency test (VFT); (ii) memory function: auditory verbal learning test of short- and long-delay free recall (AVLT-short, AVLT-long); (iii) visuospatial function: Rey-Osterrieth complex figure test (copy) (Rey-O copy); (iv) language function: Boston naming test (30 items) (BNT). The MoCA and MMSE were performed to assess overall cognitive performance. The scales have been adjusted based on each participant's educational level.

### 2.3 MRI acquisition

The MRI acquisition was performed using a 3.0 T MRI scanner in our hospital (GE Signa HDxt, USA) equipped with an 8-channel phased array head coil. Prior to the scanning of research sequences, all participants underwent a scanning of conventional sequences, including T1-weighted (T1w) imaging, T2w imaging, T2-fluid attenuated inversion recovery imaging, diffusion weighted imaging, susceptibility-weighted imaging and time-of-flight magnetic resonance angiography. Two experienced neuroradiologists were tasked with evaluating the MRI images. Once a participant satisfied the criteria, we proceeded with scanning the research sequences.

Diffusion tensor imaging (DTI) images were acquired using a spin-echo single-shot echo-planar pulse sequence (TE = 89.8 ms, TR = 17,000 ms, matrix = 128 × 128, FOV = 256 × 256 mm^2^, gap = 0, slice thickness = 2 mm, slices = 66, 20 diffusion-weighted directions for b-values of 1,000 s/mm^2^ and 0, respectively). Sagittal T1w images were obtained using a 3D-SPGR sequence (TE = 1.7 ms, TR = 5.5 ms, TI = 450 ms, FA = 15°, matrix = 256 × 256, FOV = 256 × 256 mm^2^, gap = 0, slice thickness = 1.0 mm, and slices = 155) for volumetric and registration purposes.

### 2.4 Image processing and analysis

#### 2.4.1 Free-water imaging

Before image processing, two experienced neuroradiologists will screen the data and exclude those with artifacts. The DTI data preprocessing was conducted using FLIRT, a component of the FMRIB Software Library (FSL; Oxford Centre for Functional MRI of the Brain, Oxford, UK; www.fmrib.ox.ac.uk/fsl). Each brain dataset underwent corrections for susceptibility-induced geometric distortions, eddy current distortions, and inter-volume motion artifacts. Concurrently, the gradient directions were adjusted in accordance with the eddy current corrections. Following these steps, FW maps were calculated from the corrected volumes through open-source software (https://github.com/sameerd/DiffusionTensorImaging) as previously described (Chen et al., 2023). FW maps were generated through the application of a bi-tensor model, which was informed by the diffusion measurements. This bi-tensor model estimates the signal attenuation attributable to both intracellular and extracellular water. Given that free water is predominantly located in the extracellular space, the FW maps effectively quantify the volumetric fraction of free water content within each voxel.

#### 2.4.2 Image registration and label generation

**Figure 1:**
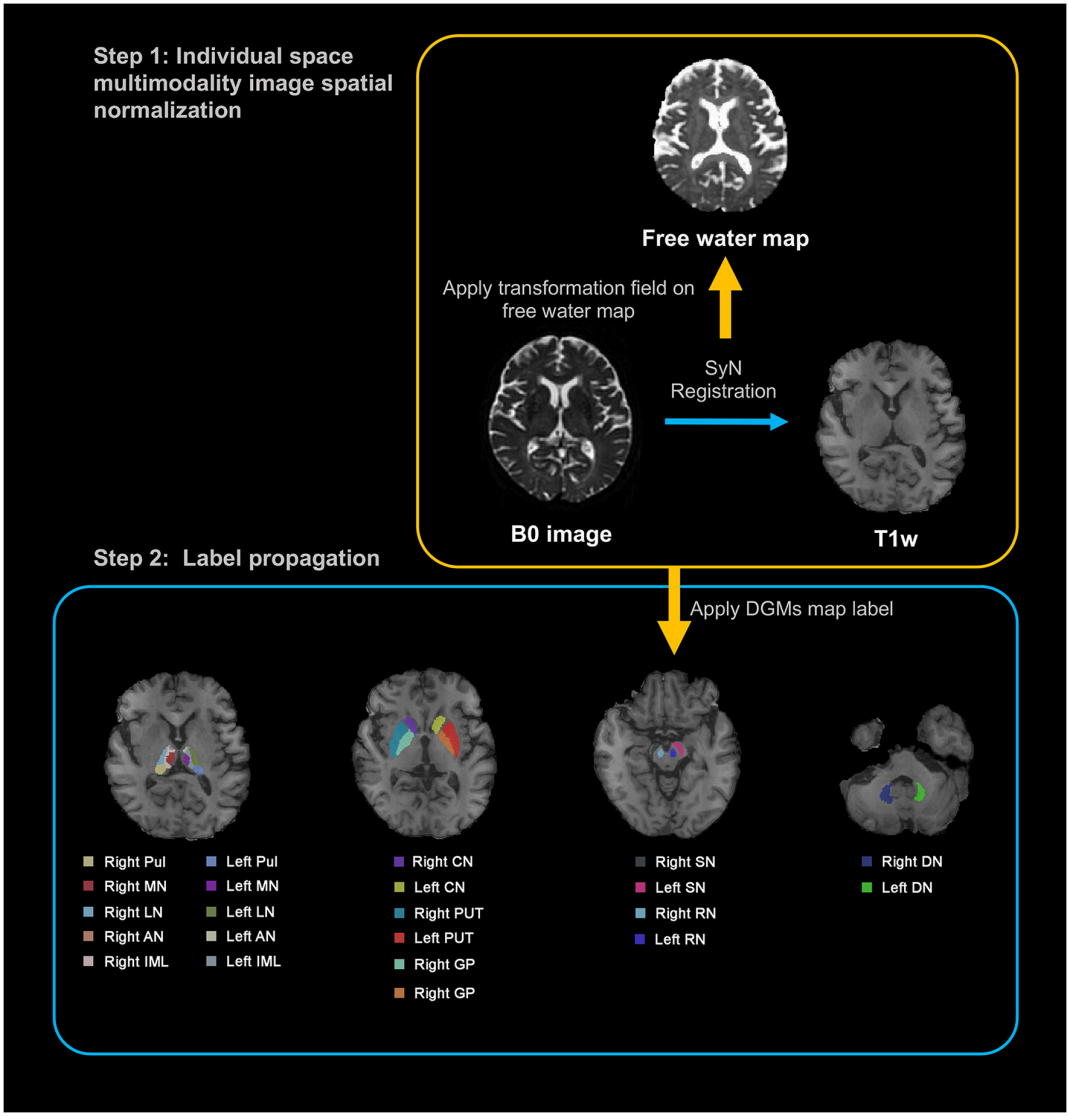
Illustration of a processing pipeline of image registration and generation of labels. T1w, T1-weighted; DGM, deep gray matter; Pul, pulvinar nucleus of the thalamus; MN, median nuclei of the thalamus; LN, lateral nuclei of the thalamus; AN, anterior nuclei of the thalamus; IML, internal medullary lamina of the thalamus; CN, caudate nucleus; PUT, putamen; GP, globus pallidus; SN, substantia nigra; RN, red nucleus; DN, dentate nucleus.

Figure 1 delineates the workflow for image registration and label generation. We used the T1-weighted (T1w) images as the intermedium for unifying the multimodal images for each participant, and to lead Region of Interest (ROI) annotations from Montreal Neurological Institute (MNI) standard space to individual image space. For each participant, the B0 image was aligned to the corresponding T1w image via SyN registration. The resulting deformation field was subsequently employed to register FW map to the T1w image. In the second phase, individual T1w images were registered to the MNI standard space using SyN registration (Avants et al., 2008). The inverse deformation field was applied to map the DGM labels, as defined by Zhang et al. (2018), from MNI standard space to the native T1w space of each participant. Notably, this DGM map was crafted using an age-specific (60–70 years) quantitative susceptibility mapping template situated in MNI space, chosen for its exceptional contrast between DGM structures and surrounding tissue (Zhang et al., 2018). This map has been previously employed in a range of neurodegenerative disease studies (Pontillo et al., 2021; Zhou et al., 2021). Two experienced neuroradiologists checked the data to ensure the accuracy of ROI regions. Following the comprehensive registration of multimodal images, we computed the average values within each predefined ROI across all imaging modalities to facilitate subsequent statistical analysis. We chose the bilateral caudate nucleus (CN), globus pallidus (GP), putamen (PUT), substantia nigra (SN), red nucleus (RN), and dentate nucleus (DN) as the ROI for analysis. Furthermore, we selected the thalamic subregions, which include the bilateral anterior nuclei (AN), the median nuclei (MN), the lateral nuclei (LN), the pulvinar (Pul), and the internal medullary lamina (IML) of the thalamus for investigation.

### 2.5 Statistical analyses

Statistical analyses were conducted using SPSS (IBM SPSS Statistics version 23.0; IBM Corporation, Armonk, NY, USA). Kolmogorov-Smirnov method was used to test the normality the indicators before group comparison. Sex of the participants were compared using chi-squared test. Age and education of the participants were compared using independent *t*-test and one-way ANOVA. ANCOVA with the covariates of age, sex and education was applied for comparison of FW to assess cognition-related differences among VaMCI, NCI and HC. *Post-hoc t*-tests were performed for pairwise comparisons of FW between groups, with age, sex and education serving as covariates. The false discovery rate (FDR) method was utilized, and a *q*-value threshold for < 0.05 (after correction) was considered statistically significant. In longitudinal study, a mixed factorial ANOVA was performed. Baseline and follow-up FW values were compared and tested for (i) the effects of time (baseline vs. follow-up), (ii) study group (VaMCI vs. NCI), and (iii) the interaction effect of time by disease. *p* < 0.05 was considered statistically significant in the longitudinal part of our study (without FDR correction as an explorative study). In addition, specific within-study-group time effects were tested using the paired *t*-test, and between-study-group baseline and follow-up differences were tested using the independent *t-*test. Effect sizes were estimated using Hedges' *g*.

In longitudinal study, the values were extracted from the DGM exhibiting significant differences in MRI indices. Delta values were calculated as the degree of change between the follow-up and baseline (ΔFW_followup − baseline_/FW_baseline_) in the VaMCI and NCI groups. Partial correlation analyses were performed to assess whether longitudinal MRI index changes (e.g., ΔFW_followup − baseline_/FW_baseline_) were associated with cognitive function changes (e.g., ΔMoCA_followup − baseline_/MoCA_baseline_) over time, with age, sex, years of education and mean follow-up time as covariates. The results were considered statistically significant when *p* < 0.05.

## 3 Results

### 3.1 Demographic and clinical information

Table 1 summarizes the demographic and clinical characteristics of the three groups—VaMCI, NCI, and HC. The baseline evaluation indicated no significant difference in age or sex among the three groups (103 VaMCI patients, 67 NCI patients, and 21 HCs). The results demonstrated that the VaMCI group had significantly fewer years of education compared with both the NCI and HC groups (*p* < 0.05). In addition, there was no difference in the proportion of risk factors among participants from VaMCI and NCI. Of the 51 SVD participants included in the follow-up, both the 28 VaMCI and 23 NCI groups exhibited similar age brackets, sex distribution, and education levels at baseline. However, diabetes mellitus appeared more frequently among VaMCI participants than NCI participants. No other risk factors showed any prevalent difference between the groups. The mean follow-up time was 1.19 ± 0.47 years for the VaMCI group and 1.17 ± 0.37 years for the NCI group.

**Table 1:** Demographic and clinical characteristics of the participants.

	**VaMCI (*n* = 103)**	**NCI (*n* = 67)**	**HCs (*n* = 21)**	***p*-values**	**VaMCI (*n* = 28)^i^**	**NCI (*n* = 23)^i^**	***p*-values^i^**
**Demographic factors**							
Age (mean ± SD)	65.17 ± 6.65	64.37 ± 7.06	61.95 ± 5.15	0.130^a^	65.89 ± 6.31	63.22 ± 6.63	0.147^b^
Sex (male/female)	76/27	57/10	17/4	0.207^c^	24/4	19/4	0.762^c^
Education years (mean ± SD)	10.07 ± 2.73	11.66 ± 2.94	11.71 ± 1.62	< 0.001^a,*,**^	9.82 ± 2.00	10.43 ± 2.39	0.323^b^
**Risk factors**							
Hypertension (%)	73.79%	68.66%	/	0.468^c^	82.14%	73.91%	0.477^c^
Diabetes mellitus (%)	35.92%	25.37%	/	0.149^c^	50.00%	21.74%	**0.038** ^c^
Hyperlipidemia (%)	14.56%	10.45%	/	0.435^c^	17.86%	8.70%	0.344^c^
Cigarette smoking (%)	45.63%	58.21%	/	0.109^c^	42.86%	60.87%	0.200^c^
Mean follow-up time in years (mean ± SD)	/	/	/	/	1.19 ± 0.47	1.17 ± 0.37	0.825^b^

VaMCI, vascular mild cognitive impairment; NCI, no cognitive impairment; HCs, healthy controls; SD, standard deviation.

^i^Demographic and clinical characteristics at baseline of the participants who were followed up.

^a^One-way ANOVA.

^b^Independent *t*-test.

^c^Chi-squared test.

^*^Represents a significant difference between VaMCI and HCs.

^**^Represents a significant difference between VaMCI and NCI.

*p*-value < 0.05 was considered statistically significant (highlighted in bold and italics).

Table 2 shows the neuropsychological characteristics of the VaMCI, NCI and HCs in cross-sectional group comparisons. A significant main effect of diagnosis (*p* < 0.001) was observed on overall cognitive performance. Notably, the VaMCI group exhibited lower attention, executive function, memory, language and visuospatial capabilities relative to the NCI group.

**Table 2:** Neuropsychological characteristics of the cross-sectional group comparisons.

	**VaMCI (*n* = 103)**	**NCI (*n* = 67)**	**HCs (*n* = 21)**	***p*-values^b^**
**Overall cognitive performance**				
MoCA	21.40 ± 3.67	26.91 ± 1.45	28.62 ± 0.86	< 0.001^a*,**,***^
MMSE	26.56 ± 1.92	28.52 ± 1.28	28.71 ± 0.90	< 0.001^a*,**^
**Attention and executive function**				
TMT-A	99.50 ± 44.92	57.69 ± 18.19	/	*** < 0.001** ^***b***^*
TMT-B	244.20 ± 99.75	143.94 ± 43.10	/	*** < 0.001** ^***b***^*
Stroop C-T	124.86 ± 52.83	80.96 ± 15.41	/	*** < 0.001** ^***b***^*
VFT	13.12 ± 3.90	16.22 ± 3.60	/	*** < 0.001** ^***b***^*
**Memory function**				
AVLT-short	3.87 ± 1.99	6.60 ± 2.01	/	*** < 0.001** ^***b***^*
AVLT-long	3.03 ± 2.06	6.36 ± 2.29	/	*** < 0.001** ^***b***^*
**Language function**				
BNT	21.58 ± 3.61	25.70 ± 2.50	/	*** < 0.001** ^***b***^*
**Visuospatial function**				
Rey-O copy	31.62 ± 5.99	35.10 ± 1.53	/	*** < 0.00** ^***b***^*

VaMCI, vascular mild cognitive impairment; NCI, no cognitive impairment; HCs, healthy controls; MoCA, Montreal cognitive assessment; MMSE, mini mental state examination; TMT-A, trail-making tests A; TMT-B, trail-making tests B; Stroop C-T, Stroop color-word test C; VFT, category verbal fluency test; AVLT-short, Rey auditory verbal learning test of short-delay free recall; AVLT-long, Rey auditory verbal learning test of long-delay free recall; BNT, Boston naming test (30 items); Rey-O copy, Rey-Osterrieth complex figure test (copy).

^a^One-way ANOVA.

^b^Independent *t*-test.

^*^Represents a significant difference between VaMCI and HCs.

^**^Represents a significant difference between VaMCI and NCI.

^***^Represents significant difference between NCI and HCs.

*p*-value < 0.05 was considered statistically significant (highlighted in bold and italics).

For longitudinal insights, Table 3 presents the neuropsychological characteristics of the 28 VaMCI and 23 NCI patients. The VaMCI group had lower overall cognitive performance, attention, executive function, and memory, both at baseline and during follow-up. However, the groups showed no significant differences in language or visuospatial functions. No significant differences appeared in any cognitive tests between the baseline and follow-up for VaMCI patients. In contrast, the NCI group demonstrated improved attention and executive function at follow-up compared with baseline.

**Table 3:** Neuropsychological characteristics in longitudinal study.

	**Baseline**		**Follow-up**		***p*-values^a^**	***p*-values^b^**	***p*-values^c^**	***p*-values^d^**
	**VaMCI (*****n*** = **28)**	**NCI (*****n*** = **23)**	**VaMCI (*****n*** = **28)**	**NCI (*****n*** = **23)**				
**Overall cognitive performance**								
MoCA	22.39 ± 3.55	26.26 ± 1.76	22.21 ± 3.71	25.22 ± 3.71	*** < 0.001***	***0.001***	0.747	0.229
MMSE	27.07 ± 1.98	28.57 ± 1.67	26.82 ± 3.08	28.30 ± 1.61	***0.006***	***0.042***	0.067	0.479
**Attention and executive function**								
TMT-A	91.43 ± 44.35	70.43 ± 25.29	94.43 ± 60.75	55.65 ± 15.37	***0.040***	***0.003***	0.731	***0.004***
TMT-B	215.04 ± 82.10	157.26 ± 55.39	205.36 ± 77.20	136.17 ± 34.86	***0.006***	*** < 0.001***	0.330	***0.012***
Stroop C-T	122.50 ± 53.13	93.74 ± 42.45	122.89 ± 48.71	77.52 ± 16.76	***0.041***	*** < 0.001***	0.966	***0.028***
VFT	14.11 ± 3.87	15.17 ± 2.98	12.68 ± 4.14	16.35 ± 2.77	0.284	*** < 0.001***	0.055	***0.036***
**Memory function**								
AVLT-short	4.36 ± 1.83	5.91 ± 2.02	4.46 ± 2.35	6.57 ± 1.53	***0.006***	*** < 0.001***	0.807	0.061
AVLT-long	3.18 ± 2.07	5.74 ± 2.77	3.75 ± 2.68	7.30 ± 6.05	*** < 0.001***	***0.007***	0.226	0.133
**Language function**								
BNT	22.79 ± 3.57	24.48 ± 2.98	23.29 ± 4.26	24.43 ± 3.01	0.076	0.282	0.402	0.929
**Visuospatial function**								
Rey-O copy	32.21 ± 4.11	34.04 ± 3.59	32.14 ± 5.81	33.87 ± 4.97	0.101	0.265	0.950	0.899

NCI, no cognitive impairment; VaMCI, vascular mild cognitive impairment; MoCA, Montreal cognitive assessment; MMSE, mini mental state examination; TMT-A, trail-making tests A; TMT-B, trail-making tests B; Stroop C-T, Stroop color-word test C; VFT, category verbal fluency test; AVLT-short, Rey auditory verbal learning test of short-delay free recall; AVLT-long, Rey auditory verbal learning test of long-delay free recall; BNT, Boston naming test (30 items); Rey-O copy, Rey-Osterrieth complex figure test (copy).

^a^*p*-value of the difference between VaMCI and NCI at baseline (independent *t*-test).

^b^*p*-value of the difference between VaMCI and NCI at follow-up (independent *t*-test).

^c^*p*-value of the group difference in VaMCI between baseline and follow-up (paired *t*-test).

^d^ *p*-value of the group difference in NCI between baseline and follow-up (paired *t*-test).

*p*-value < 0.05 was considered statistically significant (highlighted in bold and italics).

### 3.2 Group difference in free water values in deep gray matter

#### 3.2.1 Cross-sectional results of free water values in deep gray matter

**Figure 2:**
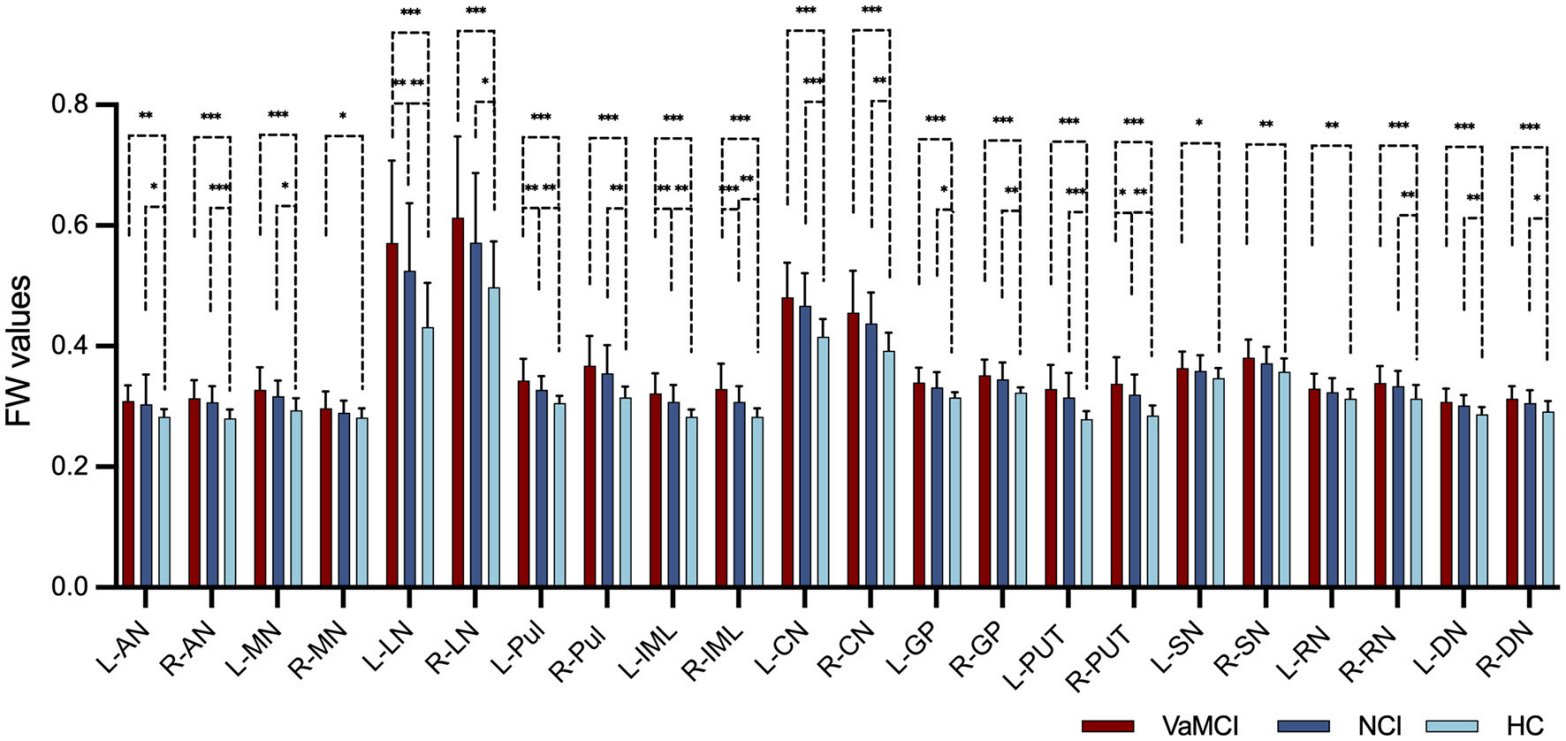
FW values in the DGM among VaMCI, NCI and HC groups in cross-sectional group comparison. Significant differences in the mean FW values across all the DGM regions among the three participant groups were represented as *q* < 0.05 FDR-corrected, following an ascending pattern (FW values: HC < NCI < VaMCI). Statistically significant difference in the *post-hoc t*-tests is delineated by black solid bracket. *Represents *q* < 0.05; **represents *q* < 0.01; ***represents *q* < 0.001. FW, free water; DGM, deep gray matter; VaMCI, vascular mild cognitive impairment; NCI, no cognitive impairment; HCs, healthy controls; L, left; R, right; AN, anterior nuclei of the thalamus; MN, median nuclei of the thalamus; LN, lateral nuclei of the thalamus; Pul, pulvinar nucleus of the thalamus; IML, internal medullary lamina of the thalamus; CN, caudate nucleus; GP, globus pallidus; PUT, putamen; SN, substantia nigra; RN, red nucleus; DN, dentate nucleus.

Table 4 and Figure 2 illustrate the distribution of FW values in DGM for the VaMCI, NCI and HC groups at baseline. The results revealed significant differences in the mean FW values across all the DGM regions among the three participant groups (all *q* < 0.05 FDR-corrected, FW values: HC < NCI < VaMCI). In VaMCI patients, significantly higher FW values were observed in DGM compared to HCs. Similarly, in NCI patients, most DGM regions exhibited significantly higher FW values than in HCs. Compared VaMCI to NCI, the study identified a significant increased FW values in VaMCI at the left LN of the thalamus (*q* = 0.004), left Pul of the thalamus (*q* = 0.004), bilateral IML of the thalamus (left: *q* = 0.004; right: *q* < 0.001) and right PUT (*q* = 0.010).

**Table 4:** Cross-sectional results of free water values in deep gray matter.

	**VaMCI (*n* = 103)**	**NCI (*n* = 67)**	**HCs (*n* = 21)**	***q*-values^a^**	**VaMCI vs. HCs**	**NCI vs. HCs**	**VaMCI vs. NCI**
L-anterior nuclei of thalamus	0.309 ± 0.026	0.304 ± 0.049	0.283 ± 0.013	***0.016***	***0.005***	***0.044***	0.623
R-anterior nuclei of thalamus	0.314 ± 0.030	0.307 ± 0.027	0.280 ± 0.015	*** < 0.001***	*** < 0.001***	*** < 0.001***	0.263
L-median nuclei of thalamus	0.328 ± 0.037	0.317 ± 0.026	0.294 ± 0.020	*** < 0.001***	*** < 0.001***	***0.012***	0.065
R-median nuclei of thalamus	0.297 ± 0.028	0.290 ± 0.020	0.282 ± 0.015	***0.025***	***0.023***	0.380	0.137
L-lateral nuclei of thalamus	0.571 ± 0.137	0.525 ± 0.112	0.432 ± 0.073	*** < 0.001***	*** < 0.001***	***0.007***	***0.004***
R-lateral nuclei of thalamus	0.613 ± 0.135	0.572 ± 0.115	0.498 ± 0.076	***0.001***	*** < 0.001***	***0.043***	0.095
L-pulvinar of thalamus	0.343 ± 0.036	0.328 ± 0.022	0.306 ± 0.012	*** < 0.001***	*** < 0.001***	***0.009***	***0.004***
R-pulvinar of thalamus	0.368 ± 0.049	0.355 ± 0.047	0.315 ± 0.018	*** < 0.001***	*** < 0.001***	***0.002***	0.149
L-internal medullary lamina of thalamus	0.322 ± 0.033	0.308 ± 0.028	0.283 ± 0.012	*** < 0.001***	*** < 0.001***	***0.002***	***0.004***
R-internal medullary lamina of thalamus	0.329 ± 0.042	0.308 ± 0.026	0.283 ± 0.014	*** < 0.001***	*** < 0.001***	***0.010***	*** < 0.001***
L-caudate nucleus	0.481 ± 0.057	0.467 ± 0.054	0.416 ± 0.029	*** < 0.001***	*** < 0.001***	*** < 0.001***	0.251
R-caudate nucleus	0.456 ± 0.069	0.438 ± 0.051	0.392 ± 0.030	*** < 0.001***	*** < 0.001***	***0.006***	0.150
L-globus pallidus	0.340 ± 0.024	0.332 ± 0.025	0.315 ± 0.009	*** < 0.001***	*** < 0.001***	***0.011***	0.100
R-globus pallidus	0.352 ± 0.026	0.345 ± 0.028	0.323 ± 0.009	*** < 0.001***	*** < 0.001***	***0.002***	0.172
L-putamen	0.329 ± 0.040	0.315 ± 0.041	0.279 ± 0.013	*** < 0.001***	*** < 0.001***	*** < 0.001***	0.064
R-putamen	0.338 ± 0.044	0.320 ± 0.033	0.285 ± 0.017	*** < 0.001***	*** < 0.001***	***0.001***	***0.010***
L-substantia nigra	0.364 ± 0.027	0.359 ± 0.026	0.347 ± 0.017	***0.034***	***0.018***	0.156	0.441
R-substantia nigra	0.381 ± 0.030	0.372 ± 0.027	0.358 ± 0.022	***0.006***	***0.003***	0.120	0.135
L-red nucleus	0.330 ± 0.024	0.324 ± 0.023	0.313 ± 0.016	***0.009***	***0.004***	0.115	0.197
R-red nucleus	0.339 ± 0.028	0.334 ± 0.025	0.313 ± 0.023	***0.001***	*** < 0.001***	***0.004***	0.485
L-dentate nucleus	0.308 ± 0.022	0.302 ± 0.017	0.287 ± 0.012	*** < 0.001***	*** < 0.001***	***0.008***	0.090
R-dentate nucleus	0.313 ± 0.021	0.306 ± 0.021	0.292 ± 0.017	*** < 0.001***	*** < 0.001***	***0.017***	0.055

VaMCI, vascular mild cognitive impairment; NCI, no cognitive impairment; HCs, healthy controls; L, left; R, right.

^a^*q*-value of the cross-sectional group comparisons (one-way ANOVA, FDR corrected). Results are described as mean ± SD.

*q*-value < 0.05 was considered statistically significant (highlighted in bold and italics).

#### 3.2.2 Longitudinal results of free water in deep gray matter

**Figure 3:**
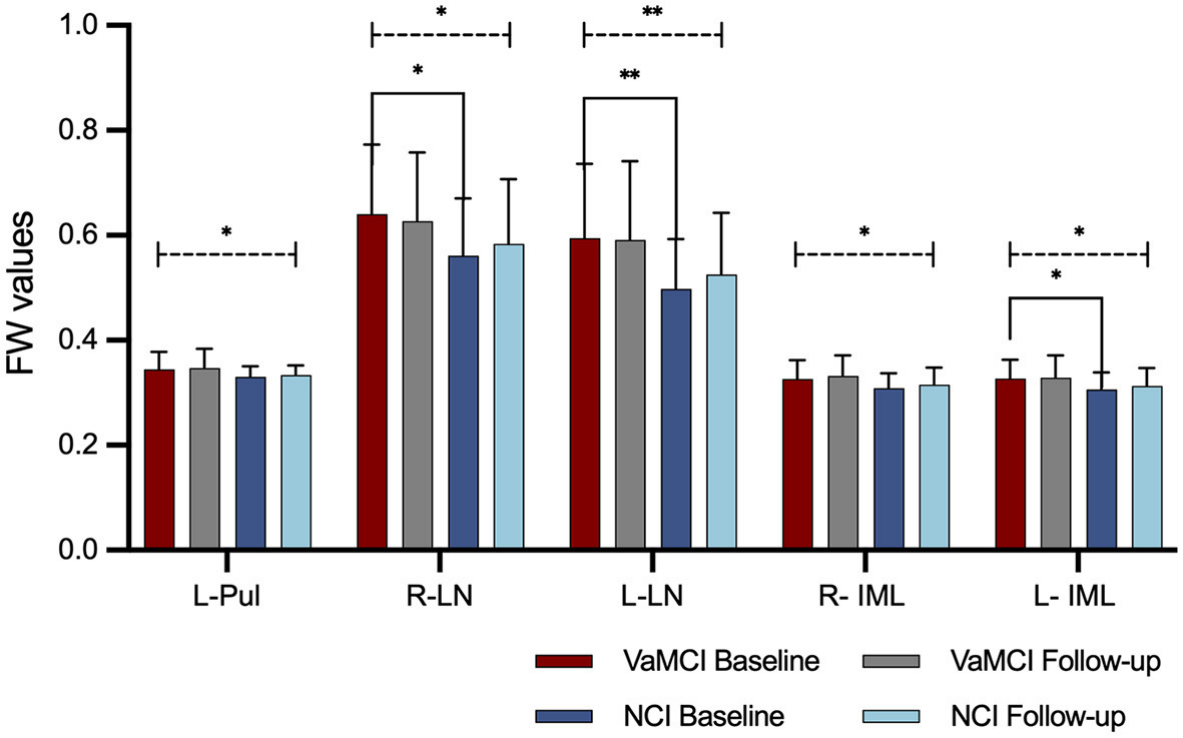
FW values in the DGM in longitudinal group comparisons. A group effect resulted in higher mean FW values within thalamic subregions for the VaMCI group as compared to the NCI group (statistically significant difference is delineated by black dashed line. *Represents *p* < 0.05; **represents *p* < 0.01). No significant effects were observed for FW values across time or group × time interactions. At baseline, FW values of the bilateral LN of the thalamus and left IML of the thalamus were significantly higher in the VaMCI group than in the NCI group (statistically significant difference is delineated by black solid bracket. *Represents *p* < 0.05; **represents *p* < 0.01). FW, free water; DGM, deep gray matter; VaMCI, vascular mild cognitive impairment; NCI, no cognitive impairment; LN, lateral nuclei of the thalamus; IML, internal medullary lamina of the thalamus; Pul, pulvinar nucleus of the thalamus; L, left; R, right.

Table 5 and Figure 3 summarize the results of the longitudinal comparison. The results detect a pronounced group effect, revealing increased mean FW values in VaMCI relative NCI, specifically in the left Pul of the thalamus (*p* = 0.037), bilateral LN of the thalamus (right: *p* = 0.015; left: *p* = 0.003) and bilateral IML of the thalamus (right: *p* = 0.015; left: *p* = 0.020). At baseline, FW values of the bilateral LN of the thalamus (right: *g* = 0.634, *p* = 0.027; left: *g* = 0.779, *p* = 0.007) and left IML of the thalamus (*g* = 0.580, *p* = 0.041) were significantly higher in the VaMCI group than in the NCI group. Notably, the follow-up data do not show any significant differences between VaMCI and NCI groups. The study also finds an absence of significant effects on the FW values across time or group × time interactions (*p* > 0.05).

**Table 5:** Longitudinal results of free water values in deep gray matter.

		**Baseline**	**Follow-up**	**Difference (effect size)**	***p* ^a^**	***p* ^c^**	***p* ^d^**
L-Pulvinar of thalamus	NCI	0.330 ± 0.020	0.334 ± 0.018	+0.004 (*g* = 0.243)	0.421	0.475	0.803
	VaMCI	0.345 ± 0.033	0.347 ± 0.037	+0.002 (*g* = 0.065)	0.762		
	Difference (effect size)	+0.015 (*g* = 0.533)	+0.013 (*g* = 0.419)				
	*p* ^b^	0.060	0.137				
	*p* ^e^	***0.037***					
R-Lateral nucleus of thalamus	NCI	0.561 ± 0.109	0.584 ± 0.123	+0.023 (*g* = 0.191)	0.528	0.853	0.485
	VaMCI	0.640 ± 0.133	0.627 ± 0.131	−0.013 (*g* = −0.098)	0.716		
	Difference (effect size)	+0.079 (*g* = 0.634)	+0.043 (*g* = 0.334)				
	*p* ^b^	***0.027***	0.234				
	*p* ^e^	***0.015***					
L-Lateral nucleus of thalamus	NCI	0.498 ± 0.095	0.525 ± 0.118	+0.027 (*g* = 0.243)	0.405	0.673	0.531
	VaMCI	0.595 ± 0.141	0.591 ± 0.150	−0.004 (*g* = −0.035)	0.892		
	Difference (effect size)	+0.097 (*g* = 0.779)	+0.066 (*g* = 0.471)				
	*p* ^b^	***0.007***	0.095				
	*p* ^e^	***0.003***					
R- Internal medullary lamina of thalamus	NCI	0.309 ± 0.028	0.315 ± 0.033	+0.006 (*g* = 0.182)	0.561	0.385	0.974
	VaMCI	0.326 ± 0.036	0.332 ± 0.039	+0.006 (*g* = 0.161)	0.516		
	Difference (effect size)	+0.017 (*g* = 0.529)	+0.017 (*g* = 0.480)				
	*p* ^b^	0.062	0.089				
	*p* ^e^	***0.015***					
L- Internal medullary lamina of thalamus	NCI	0.306 ± 0.033	0.313 ± 0.034	+0.007 (*g* = 0.204)	0.502	0.533	0.724
	VaMCI	0.327 ± 0.036	0.329 ± 0.042	+0.002 (*g* = 0.050)	0.844		
	Difference (effect size)	+0.021 (*g* = 0.580)	+0.016 (*g* = 0.390)				
	*p* ^b^	***0.041***	0.166				
	*p* ^e^	***0.020***					

NCI, no cognitive impairment; VaMCI, vascular mild cognitive impairment; L, left; R, right; Between-subject *p*-values are presented below descriptive results, while within-subject *p*-values are presented to the right of descriptive results. Results described as mean ± SD. Effect sizes were computed using Hedges' *g*.

^a^*p*-value of the within group difference between baseline and follow-up (paired *t*-test).

^b^*p*-value of the difference between VaMCI and NCI (independent *t*-test).

^c^Main effect of time (mixed factorial ANOVA).

^d^*p*-value of the interaction effect of time by group (mixed factorial ANOVA).

^e^Main effect of group (mixed factorial ANOVA).

*p*-value < 0.05 was considered statistically significant (highlighted in bold and italics).

### 3.3 Correlations between longitudinal free water value changes and cognitive function changes

**Figure 4:**
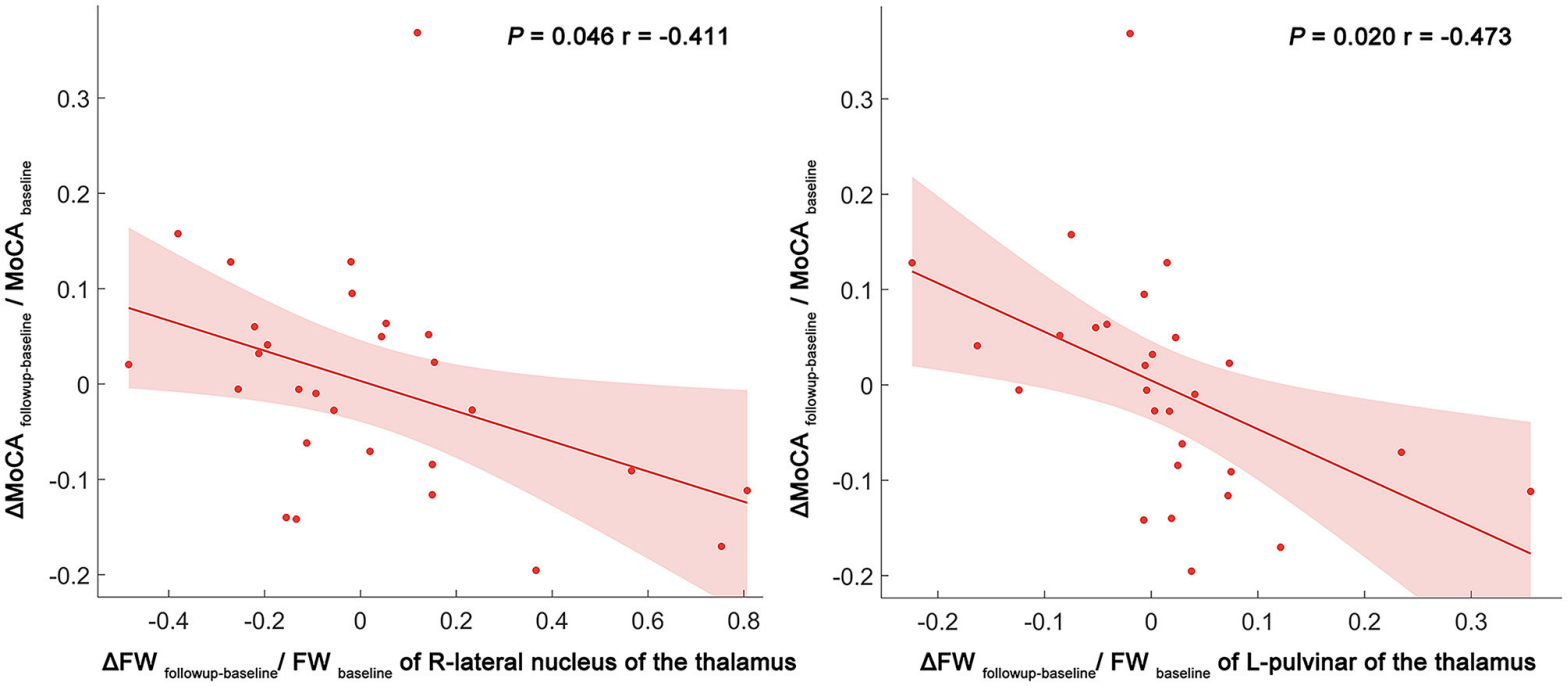
Scatter plot and linear regression illustrating the relationship between longitudinal FW changes in the right LN and left Pul, and MoCA score changes. Partial correlation analyses were performed to assess whether longitudinal FW changes were associated with cognitive function changes over time. Delta values were calculated as the degree of change between the follow-up and baseline (e.g., ΔFW_followup − baseline_/FW_baseline_) in the VaMCI and NCI groups. The dots represent the adjusted values after controlling age, sex, years of education, and mean follow-up time. FW, free water; LN, lateral nuclei of the thalamus; Pul, pulvinar nucleus of the thalamus; MoCA, Montreal cognitive assessment; FW, free water; R, right; L, left.

Over a period of 1–2 years, this study identifies negative correlations between the alterations in the FW values (calculated as: ΔFW_follow − up − baseline_/FW_baseline_) and changes in MoCA scores (calculated asΔMoCA_follow − up − baseline_/MoCA_baseline_) in the VaMCI group. These correlations manifest in the right LN of the thalamus (*p* = 0.046, *r* = −0.411) and the left Pul of the thalamus (*p* = 0.020, *r* = −0.473), with age, sex, education years and mean follow-up time as covariates. In contrast, no significant correlations were identified in the NCI group (Figure 4).

## 4 Discussion

In this longitudinal study, we utilized FW imaging to assess microstructural changes in the DGM of VaMCI, NCI, and HC groups. Three main findings emerged from our analysis. First, cross-sectional comparisons highlighted significant differences in FW values across the three participant groups, following an ascending pattern. Second, the longitudinal assessment revealed a group effect resulted in higher mean FW values within thalamic subregions for the VaMCI group as compared to the NCI group; however, no significant effects were observed for FW values across time or group × time interactions. Third, a significant negative association was observed between the FW value changes in the Pul and the LN of the thalamus, and MoCA score changes in the VaMCI group over 1–2 years. Collectively, these findings indicated the potential of FW as an imaging biomarker during the early stages of SVD.

Compelling evidence indicates that SVD pathology initiates years before clinical SVaD diagnosis, featuring a protracted preclinical or early clinical phase marked by conditions like NCI and VaMCI (Wardlaw et al., 2013a, 2019). In line with previous studies (Low et al., 2019; Evans et al., 2021), our study detected increased FW in DGM of early-stage SVD patients compared to HCs. In the healthy brain, neuronal, vascular, and inflammatory cells collaboratively sustain normal function. However, disrupted interactions among these cells trigger chronic pathological neuroinflammation. Certain risk factors for SVD, such as hypertension and diabetes mellitus, impair cerebral blood flow, damage the BBB, and initiate vascular inflammation. This BBB compromise allows fibrinogen and fluid to leak into the parenchyma, subsequently inducing edema, oxidative stress, and cognitive decline in SVD patients (Evans et al., 2021). Hypertension was identified as the most prevalent risk factor for SVD in our study, which aligns with previous research findings. It potentially plays an important role in the onset/progression of SVD (Meissner, 2016). Diabetes mellitus is also an established risk factor for CSVD (Liu et al., 2018). Diabetes mellitus patients exhibit a significantly greater degree of BBB breakdown compared to control subjects (Janelidze et al., 2017). Elevated levels of pro-inflammatory cytokines, including IL-1β, IL-6, and TNF-α, are often observed in diabetic mellitus patients (Donath and Shoelson, 2011). Therefore, diabetes mellitus probably contributes to the progression from NCI to VaMCI. Interestingly, we further discovered that diabetes mellitus occurred more frequently among VaMCI participants compared to NCI participants who participated in the longitudinal study. This observation might be linked to the higher FW values observed within thalamic subregions in the VaMCI group in the longitudinal assessment.

Conventional *in vivo* assessment of neuroinflammation remains challenging. Recently, FW imaging, leveraging a bi-tensor diffusion MRI model, offers specific and sensitive quantification of extracellular water (Pasternak et al., 2009; Metzler-Baddeley et al., 2012). This method has demonstrated superior sensitivity over traditional DTI in identifying intra- and inter-group differences (Albi et al., 2017) and has been validated as an accurate marker for neuroinflammation changes (Pasternak et al., 2009, 2012b). In addition, FW correlates with elevated brain interferon-gamma, underscoring its sensitivity to inflammation (Febo et al., 2020). Currently, it is advisable to acquire multi-shell DTI data for FW imaging in order to enhance the accuracy and comprehensiveness of the results (Nemmi et al., 2022). While multi-shell data offers greater precision, its longer acquisition time poses limitations in clinical settings. In our center, most subjects are elderly individuals who cannot endure extended MRI scans. Given the constraints in clinical settings, single-shell DTI is often preferred for its significantly shorter acquisition times, as evidenced in recent large-scale clinical studies (Andica et al., 2019; Zhou et al., 2021; Ray et al., 2023).

In all, our results extend previous findings by suggesting that even in its early stages, SVD features elevated DGM FW values, indicative of underlying neuroinflammation and DGM microstructural pathology. In addition, we found that the VaMCI group had significantly fewer years of education compared with both the NCI and HC groups. The level of education serves as a reliable indicator or proxy for cognitive reserve. Cognitive reserve refers to individual differences in how tasks are performed that may allow some people to be more resilient than others (Stern, 2012). This may be reflected in our results relating to improved attention and executive function in the NCI group longitudinally but stable cognition in the VaMCI group. Our results have taken education into account as control variables. We hope that this can initially reduce the impact of cognitive reserve on the results of our current research. Going forward, we aim to more explicitly integrate measures of cognitive reserve in neuroimaging studies of SVD.

Our longitudinal study reveals that VaMCI patients exhibit high mean FW values in the LN, Pul, and IML regions of the thalamus compared to NCI patients. Multimodal neuroimaging has already pinpointed early-stage thalamic microstructural changes in SVD, including reduced cerebral blood flow (Kato et al., 2008), axonal degeneration (Cavallari et al., 2014), and other markers such as decreased volume (Wang et al., 2020), increased diffusion (Öztoprak et al., 2015) and iron deposition (Sun et al., 2020). The thalamus, with its cortical, subcortical, and cerebellar connections, is a critical node in brain networks supporting cognitive functions known to decline in SVD, including component processes of executive functions of attention, information processing and memory (Fama and Sullivan, 2015). As such, damage to the thalamic nuclei potentially impairs a wide range of neurologic functions that may clinically translate into significant cognitive disability, which is primarily affected in the early stages of SVD (Duering et al., 2011; Bonifazi et al., 2018). However, whole-thalamic analyses fall short in capturing subregional characteristics, often because they aggregate data, diluting subtle variance at the subregional level. Thus, our study considered the structural complexity of the thalamus itself and the functional subspecialization of its nuclei. Our results indicate that elevated FW values in the LN, Pul and IML of the thalamus likely play key roles in SVD-related cognitive decline. Furthermore, FW alterations remained stable in both groups during the follow-up time. The longitudinal dimension of the current study adds a contribution to the literature, by showing that higher FW remains unchanged during the 1–2 years of VaMCI and NCI. We speculate that there could be no evidence of significant progression or remission in neuroinflammation in the early stage of SVD. Our results are consistent with previous studies showing that the inflammatory response is sustained in the long term and longitudinally associated with SVD progression (Low et al., 2019). Interestingly, we still found a trend toward increased FW in the NCI group over 1–2 years, and the NCI group showed a faster rate of increase than the VaMCI group, explaining the lack of significant FW differences between the VaMCI and NCI groups at follow-up. However, given the small sample size at follow up mandates cautious interpretation of these results.

In addition, our results also demonstrated an association between increasing FW in the Pul and LN of the thalamus and decreasing cognitive function over 1–2 years in the VaMCI group. This aligns with longitudinal study showing elevated inflammatory markers could predict SVD severity and progression (Low et al., 2019). In the realm of cognition, the Pul modulates attentional synchrony between cortical areas (Saalmann et al., 2012), while the LN act as a tunable filter serving as a cognitive control node (Halassa and Kastner, 2017). Previous studies substantiate FW as a strong MRI marker for cognitive deceleration (Berger et al., 2022) and as a disease-progression biomarker in Parkinson's disease (Ofori et al., 2015; Zhou et al., 2021). Our findings thus suggest that increased FW in the thalamus may represent a potential marker for disease course. Despite the increasing global burden of SVD and its role in dementia, established treatments remain elusive (Smith and Markus, 2020). Current interventions focus on risk management, offering limited efficacy. Given this backdrop, our study amplifies the relevance of FW values as potential neuroinflammatory marker in DGM at the preclinical and early clinical stage of SVD, offering a pathway toward targeted therapeutic interventions that may directly affect early-stage of SVD cognitive outcomes.

This study has several limitations. First, the sample size in the follow-up study was relatively small. Only 51 SVD patients with complete MRI scans and neuropsychological assessments both at baseline and at follow-up were included. The absence of healthy controls in the follow-up study further compromises our analysis. Second, our methodology describes the change in DGM over time using FW imaging but limits its scope to just two-time points. Third, the diffusion imaging data, limited to 20 directions, falls short of cutting-edge standards. Recently, the acquisition of multi-shell DTI data is recommended in FW imaging for enhanced robustness and precision. Moreover, the interpretation of single-shell DTI-FW in GM is inherently more complex and that further pathological validation is necessary to enhance our understanding of these measurements. Fourth, the impact of subtle head movements caused by cognitive dysfunction on the MRI images remains a significant concern. Fifth, FDR correction was not applied in the longitudinal part of our study as this was intended to be an exploratory analysis. Thus, future studies should aim to expand the cohort size, extend the study duration, and employ state-of-art imaging techniques to provide a more comprehensive understanding of longitudinal FW changes.

## 5 Conclusions

In summary, the present study showed that increased FW level in DGM emerge at the preclinical and early clinical stages of SVD and longitudinally remain persistent for 1–2 years before the onset of clinical deterioration. Through FW imaging, we offer an indirect measurement of neuroinflammation, leading credence to the hypothesis that diffuse neuroinflammation underpins SVD activity. Furthermore, the FW value changes within thalamic subregions could serve as an imaging biomarker for the mechanism of cognitive decline in SVD progression. Our findings may have potential implications for therapeutic interventions for SVD.

## Data availability statement

The raw data supporting the conclusions of this article will be made available by the authors, without undue reservation.

## Ethics statement

The studies involving humans were approved by the Research Ethics Committee of the Ren Ji Hospital, School of Medicine, Shanghai Jiao Tong University. The studies were conducted in accordance with the local legislation and institutional requirements. The participants provided their written informed consent to participate in this study.

## Author contributions

YS: Conceptualization, Formal analysis, Investigation, Methodology, Project administration, Writing – original draft, Writing – review & editing. XH: Formal analysis, Investigation, Methodology, Writing – review & editing. ZL: Data curation, Formal analysis, Methodology, Writing – review & editing. YQ: Formal analysis, Investigation, Methodology, Project administration, Validation, Writing – review & editing. YH: Formal analysis, Investigation, Methodology, Project administration, Visualization, Writing – review & editing. YZha: Methodology, Validation, Writing – review & editing. YD: Writing – review & editing, Writing – original draft. HW: Conceptualization, Formal analysis, Methodology, Writing – review & editing. QX: Conceptualization, Funding acquisition, Writing – review & editing. YZho: Conceptualization, Funding acquisition, Supervision, Writing – review & editing.
